# LHHW/RSM reaction rate modeling for Co-Mn/SiO_2_ nanocatalyst in Fishcher-Tropsch synthesis

**DOI:** 10.1038/s41598-024-64382-0

**Published:** 2024-06-12

**Authors:** Hamid Reza Azizi, Mohsen Mansouri, Farshad Farshchi Tabrizi, Ghobad Mansouri, Naimeh Setareshenas

**Affiliations:** 1 Department of Chemical Engineering, Ilam University, Ilam, 69315516 Iran; 2 Ilam Gas Treating Company, Tajrian, Ilam Province, Chovar Iran; 3 Department of Chemical Engineering, Shiraz University, Shiraz, 7134851154 Iran; 4 Department of Chemistry, Payame Noor University (PNU), Tehran, 19395-3697 Iran

**Keywords:** Co-Mn, Nanocatalyst, Fischer–Tropsch (FT) synthesis, Kinetic, Engineering, Chemical engineering

## Abstract

This study aims to assess the kinetics of Fischer–Tropsch (FT) reaction over the cobalt-manganese nanoparticles supported by silica oxide. Nanoparticles were synthesized by the thermal decomposition method using "[Co(NH_3_)_4_CO_3_]MnO_4_" complex and characterized by XRD, TEM, and BET techniques. The kinetics of the process were evaluated using a combination of Langmuir–Hinshelwood-Hougen-Watson (LHHW) and response surface methodology. Correlation factors of 0.9902 and 0.962 were obtained for the response surface method (RSM) and LHHW, respectively. The two methods were in good agreement, and the results showed that the rate-determining step was the reaction of the adsorbed methylene with the adsorbed hydrogen atom, and only carbon monoxide molecules were the most active species on the catalyst surface. A temperature of 502.53 K and a CO partial pressure of 2.76 bar are proposed as the optimal conditions by RSM analysis. The activation energy of CO consumption reaction was estimated to be 61.06 kJ/mol.

## Introduction

Nowadays, FTS has draws great interest as a means to produce sulfur- and nitrogen-free products by reacting mixture of hydrogen and carbon monoxide. FT synthesis sets FT products apart through the production of more linear hydrocarbons and an abundance of high alpha olefins. The liquid products resulting from FT can be purified using existing purification methods to produce high purity products, free of liquid sulfur fuels and pure chemical materials^1–4^.

Iron, cobalt, nickel, ruthenium (Ru), manganese (Mn) and rhenium (Re) serve as the most effective catalysts and promoters for FT reaction. This issue depends on the ability of metals to decompose of carbon monoxide^5^. Meanwhile, only two catalysts, iron and cobalt, have been used industrially^6,7^. An important parameter that plays a role in the selection of these two catalysts is the type of carbon feed or the source of synthesis gas used^8^. Cobalt catalysts are preferred for hydrocarbon synthesis due to their high FT activity, high selectivity for long-chain paraffins, and low activity for water–gas transfer reaction^9^. Conducting a kinetic study is vital for designing efficient and safe reactors. Table 1 presents a summary of the kinetic models for the FT reaction over cobalt-based catalysts in fixed bed reactors. For cobalt catalysts Langmuir–Hinshelwood-Hougen-Watson (LHHW) kinetic equations exhibit a divergence from their counterparts for iron catalysts, primarily due to disparities in the rate-determining step which typically involves a binary site on the surface. Moshtari et al.^10^ reported the performance and kinetic of hydrocarbon-formation reactions for a LaFe_0.7_Co_0.3_O_3_ perovskite catalyst in a fixed bed reactor. They obtained the kinetic parameters for CO consumption by developing the best-fitted model. The correlation was derived and well-fitted to experimental data using the LHHW form (according to the enol mechanism, carbon monoxide and dissociated hydrogen atoms are adsorbed and reacted on the surface of the catalyst). Niu et al.^11^ conducted the kinetics of Fischer-Trosch synthesis using an industrial cobalt-based catalyst. They stated that the rate determining step in CO activation is the hydrogenation of the dissociated C* and O* into CH* and OH*. The catalytic effect of manganese promoter on carbon monoxide breakdown arises from its electron interaction with the metal. Tucker et al. showed that the addition of manganese to cobalt-based FT catalysts increases activity and significantly improves liquid fuel efficiency by reducing carbon dioxide and methane selectivity^2^. Dinseet al. demonstrated that the increase of manganese causes a constant increase both the apparent carbon monoxide consumption rate and carbon monoxide adsorption constant in the FFT process^9^.

**Table 1:** Kinetic models of FTS based on cobalt catalyst using a fixed-bed reactor.

Catalyst	Operation conditions			Kinetic model	References
	P (Mpa)	T (°C)	H_2_/CO		
La.Fe_0.7_.Co_0.3_/γ-Al_2_O_3_	1.0–2.0	240–300	1.0–2.0	$\frac{{k}_{p}{b}_{CO}{P}_{CO}{({b}_{H2}{P}_{H2})}^{0.5}}{{(1+{b}_{CO}{P}_{CO}+{({b}_{H2}{P}_{H2})}^{0.5})}^{2}}$	^10^
Industrial cobalt based	1.0–5.6	170–215	2.0–3.0	$\frac{K{P}_{CO}^{0.5}{P}_{H2}^{0.5}}{{(1+K{P}_{CO})}^{2}}$	^11^
Co//γ-Al_2_O_3_	0.2–0.8	190–260	2.0	$\frac{K{P}_{CO}{P}_{H2}}{{(1+a{P}_{CO}+b{P}_{H2}^{0.5})}^{2}}$	^12^
Co/TiO_2_	2.0265	180–240	1–3.5	$\frac{{kP_{H2}^{0.74} P_{CO} }}{{\left( {1 + aP_{CO} } \right)^{2} }}$	^13^
Co-Ce/SiO_2_	0.1	200–300	1–3	$\frac{{kP_{{H_{2} }} }}{{\left( {1 + aP_{CO} } \right)}}$	^14^
Co-Mn	0.1–1	190–250	1–2	$\frac{{k\,P_{{{\text{CO}}}} P_{{{\text{H}}_{{2}} }} }}{{\left( {1 + aP_{{{\text{H}}_{{2}} }}^{{{0}{\text{.5}}}} + bP_{{{\text{CO}}}} P_{{{\text{H}}_{{2}} }}^{{{0}{\text{.5}}}} } \right)^{2} }}$	^15^
Co-Ni/SiO_2_	1–5	280–320	1–3	$\frac{{kP_{{H_{2} }}^{0.5} P_{{{\text{CO}}}} }}{{\left( {1 + aP_{{{\text{CO}}}} + bP_{{H_{2} }}^{0.5} + cP_{{H_{2} }}^{0.5} P_{CO} } \right)^{2} }}$	^16^
Co-Mn-Ce/SiO_2_	0.1–0.6	250–350	1–2	$\frac{{k\,P_{{{\text{CO}}}} P_{{{\text{H}}_{{2}} }} }}{{\left( {1 + aP_{{{\text{H}}_{{2}} }} + bP_{{{\text{CO}}}} } \right)^{2} }}$	^17^
Co-Re/ γ-Al_2_O_3_	2.0–2.2	210–230	1.12–2.55	$\frac{{k}_{{P}_{CO}}{P}_{H2}^{0.5}(1+{k}_{{P}_{H2}}{k}_{{P}_{H2O}})}{{(1+{\alpha }^{,}{P}_{CO}+b{P}_{H2}^{0.5}+{f}^{,}{P}_{H2O})}^{2}}$	^18^

In heterogeneous reactions, intense interaction between the base and active metal impairs both the catalysts regeneration capability and activity. However, low interaction causes the movement of active points and their agglomeration and again reduces the activity of the catalyst^19^. The most famous bases for FT catalysts are silica, titania, alumina, zirconia, zeolites, and it seems that silica is the best base for iron and cobalt catalysts because of its good activity provides^19,20^. Thus, conducting research on the performance of a SiO_2_ supported Co-Mn catalyst for FTs is advantageous.

Two methods exist for modeling and predicting FTS process data. Using the LHHW mechanistic approach, the kinetic model of carbon monoxide consumption can make accurate predictions by defining four different hypotheses: carbide, enolic, combined enolic-carbide, and parallel enolic-carbide. Each elementary reaction in these mechanisms proceeds independently at its own rate. Considering each of these steps as the slowest step in the overall reaction, the best kinetic model has been presented^13,15^. Kinetic modeling accurately represents the sequence and rate of various chemical reactions occurring at a catalyst's surface. Pordeli et al.^12^ explored the role of tin as a promoter for in enhancing kinetic parameters and mechanism of Fischer–Tropsch synthesis using Co/γ-Al_2_O_3_ as catalyst. The catalytic systems mechanism was founded on the enol reaction, with CO species adsorbed molecularly and H_2_adsorbed dissociatively or associatively on the surface. The second approachch employs the response surface method (RSM) to statistically model the relationship between process and response variables using polynominal equation. Researchers can assess the impact of independent variables and the interdependence of parameters using this method. Reaction rate modeling in FTS has received limited focus from RSM^21^. The majority of FTS studies have used the "kinetic modeling" approach without taking interactions between parameters into account. A limited number of authors have explored the usage of RSM and kinetic modeling approaches to determine and enhance operational factors in FTS^16,17^. Zohdi-Fasaei et al.^17^ utilized RSM to investigate the Co-Mn-Ce/SiO_2_ catalyst's performance. The RSM rate expression aligned with the LHHW kinetic model, according to their report.

In kinetic studying of carbon monoxide consumption in FTS, the accuracy of the equation of chemical rates increases exponentially. Obtaining data from a fixed bed micro-reactor is costly and time-consuming. The mathematical model capable of estimating experimental data holds significant value for the industry. This study aims to assess the CO hydrogenation rate on Co–Mn nanocatalyst produced via thermal decomposition of "[Co(NH_3_)_4_CO_3_]MnO_4_" and SiO_2_ support. The FTS reaction rate was optimized using the kinetic modeling of the hybrid RSM/LHHW system. The interplay and independent influences of temperature, H_2_ partial pressure, and carbon monoxide's partial pressure on the FT reaction rate were examined using experimental design and response surface techniques. Regression methods have been employed to construct and authenticate mathematical models describing the FT reaction kinetics as a function of maximum reaction variable rates. Mathematical models for the kinetics of the FT reaction have been created and confirmed using regression techniques, with a focus on the impact of variable maximum reaction rates.

## Materials and procedures

### Synthesis of cobalt and manganese metals

The nanoparticles of cobalt and manganese metals were synthesized using the thermal decomposition of"[Co(NH_3_)_4_CO_3_]MnO_4_" complex as following:

The equal amounts (60 ml) of concentrated ammonia solution (NH_3_, 25%, Merck, Germany) and ammonium carbonate ((NH_4_)_2_CO_3_, ≥ 99%, Merck, Germany) were added together and premixed for 30 min at ambient temperature. Then, 15 g of cobalt nitrate (Co (NO_3_)_2_. 6H_2_O, ≥ 98%, Merck, Germany) were added and after 60 min rigorously stirring, hydrogen peroxide (H_2_O_2_, 30%, Merck, Germany) added dropwise to solution. The mixture was concentrated by immersing in hot water bath for 45 min. The concentrated mixture was filtered and kept a night in ambient temperature to form [Co(NH_3_)_4_CO_3_]NO_3_ nono crystals with red color. After this, the amount of 13 g of [Co(NH_3_)_4_CO_3_]NO_3_ was solved in deionized water premixed with 8 g of potassium permanganate (KMnO_4_, 99%, Merck, Germany) and stirred for 15 min to produce precipitates of [Co(NH_3_)_4_CO_3_]MnO_4_ complex. The precipitate immediately filtered, washed and dried in ambient temperature for 24 h. The dried precipitate was calcined at 400 °C for 4 h. to form a spaniel and gray powder so-called Co-Mn nanoparticles.

### Co-Mn/SiO_2_ catalyst

The Co-Mn nanocatalyst for FT process was synthesized by incorporating nanoparticles into SiO_2_as support via the sol–gel method with a weight ratio of 70:30 for nanoparticles to support. Co and Mn nanoparticles were dissolved in ethanol and premixed with tetraethoxysilane (Si(OC_2_H_5_)_4_, TEOS, ≥ 99%, Merck, Germany) as a precursor for generating the three-dimensional silica-based catalyst. Nitric acid was added over a 50-min period while the mixture was being gradually stirred to ensure homogeneity. The solution was stirred for 2 h after it reached complete homogeneity to prepare the gel. The pH of the solution rose from 1 to 3 while the gel was being formed. The mixture was then dried at 120 °C for 12 h and calcined at 600 °C in an electric furnace for 6 h in the presence of air.

### Instruments

Pore volume (Vp), specific surface area (Sg), and pore size (Dp)of the samples were characterized by N_2_physisorption at − 196 °C in a Micromeritics ASAP 2020 (Micromeritics, Norcross, GA, USA) analyzer. The samples were degassed at 220 °C for 6 h before analysis. The specific surface area was calculated by the Brunauer–Emmett–Teller theory, and BJHprocedurewas employed to calculate the Dp and Vp using the desorption branch.

XRD patterns were performed on a RigakuMiniflex X-ray diffractometer with a Cu Kαradiation anode (λ = 1.5418 Å) at 15 mA and 30 kV.

The diffraction patterns were collected in the 2θrange from 10° to 60° with a step size of 0.05° per step.TEM, STEM were performed with a JEOL JEM-2100F microscope. JEOL-2100F is an advanced 200 kV TEM with a Schottky type field emission electron source. The samples were prepared by sonication of the calcined or spent catalyst in isopropyl alcohol before dripping the resultant suspension onto copper grids coated with a lacey carbon film.

### FTS tests

**Figure 1:**
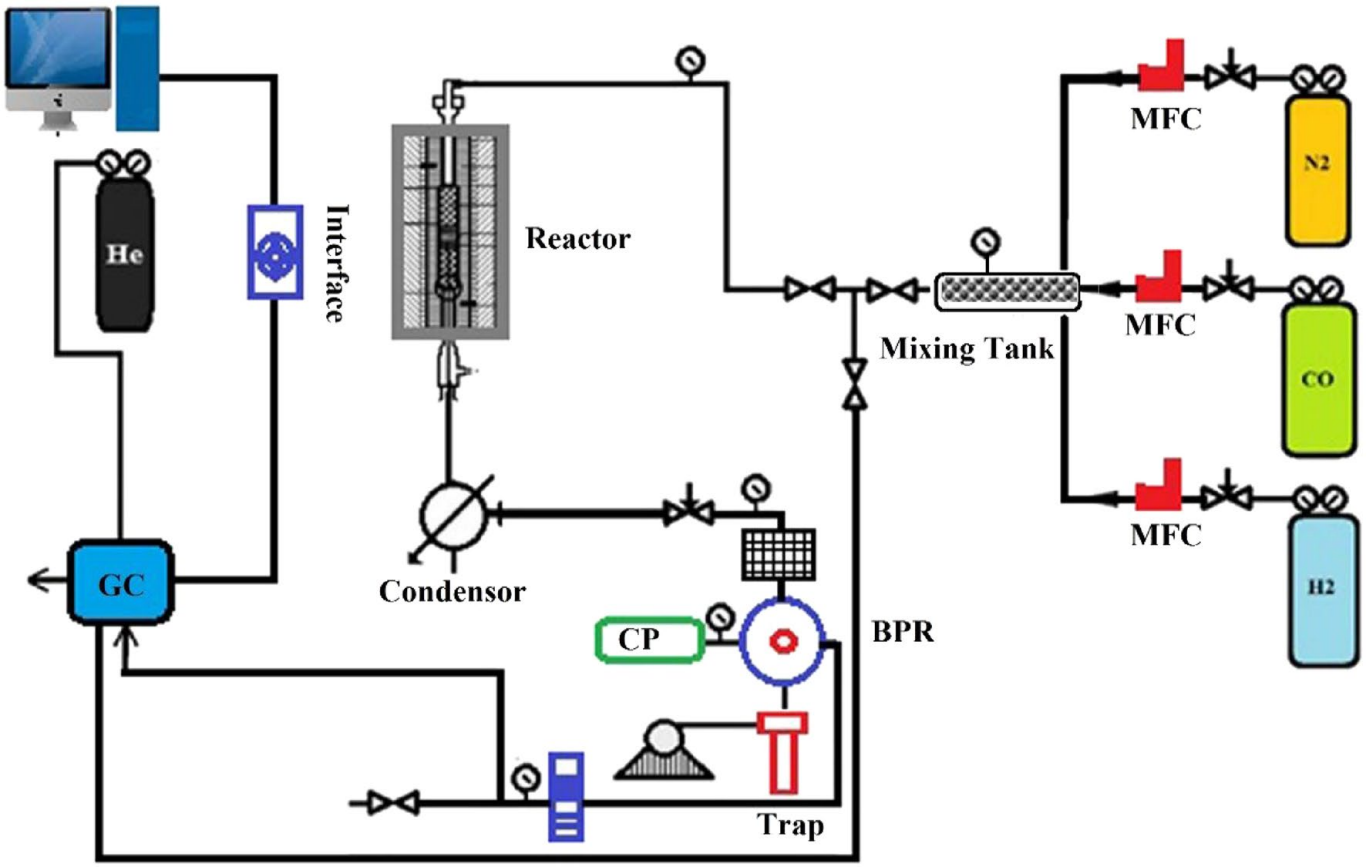
Scheme for used reactor in Fischer–Tropsch reaction testing.

A stainless steel fixed-bed micro reactor was employed to perform Fischer − Tropsch reaction. The required amount of catalyst (1 g) diluted with quartz wool was loaded in middle of the reactor. Catalyst reduction was performed at 300 °C with a heating rate of 5 °C /min for 4 h under a flow of 60 ml/minof H_2_/CO.The catalytic results were recorded at the steady state after stabilization of 12 h. To be ensure that 12 h is a sufficient time to reach steady state operation for Fischer–Tropsch using our catalyst, after reduction of catalyst and before we start the kinetic tests, we have controlled the reproducibility and consequently stabilization for the catalytic data. We tested a special FT reaction over a catalyst for 12 h. When the reaction finished, we took the used catalyst from the reactor and then put the same fresh catalyst and tested the same FT reaction again. We repeated this experiment for three times to be sure from our control and then compared the results and we found that the catalyst performance was successful and the obtained results were comparable and very close together. A view representation of the reactor is disclosed in Fig. 1.

The geometry of the reactor consisted of a 20 mm inner diameter tube encased in an alumina jacket for even wall temperature. A tubular electrical furnace was incorporated into the jacket for external heating. Three thermocouples, placed in the preheating zone, catalyst bed and underneath zone of the reactor, were utilized to measure the temperatures of various sections during the top-down feeding process. Products were analyzed using an online gas chromatograph featuring Flame Ionization and Thermal Conductivity detectors. Required data for modeling were calculated as:

1 $$X_{CO} = \left( {1 - \frac{{S_{CO.out} }}{{f_{CO} }} \times \frac{{f_{N2} }}{{S_{N2.out} }} \times \frac{{f_{CO} }}{{S_{CO.in} }} \times \frac{{S_{N2.out} }}{{f_{N2} }}} \right)$$

2 $$- r_{CO} = \frac{{X_{CO} \times v_{0} \times P_{CO}^{0} }}{w \times R \times T}$$

where S_CO,in_ and S_N2,in_ are the peak areas of carbon monoxide and nitrogen in the chromatograms of the inlet, S_CO,out_ and S_N2,out_ are the peak areas of the respective gases in the chromatograms of the outlet, and $f_{CO}$ and $f_{N2}$ are their response factors, $v_{0}$ is is volumetric gas flow rate (ml/min), $P_{CO}^{0}$ is carbon monoxide partial pressure (bar), R is universal gas constant (ml bar/kmol), w is catalyst weight (g) and T is reaction temperature (K).

### Experimental design

**Figure 2:**
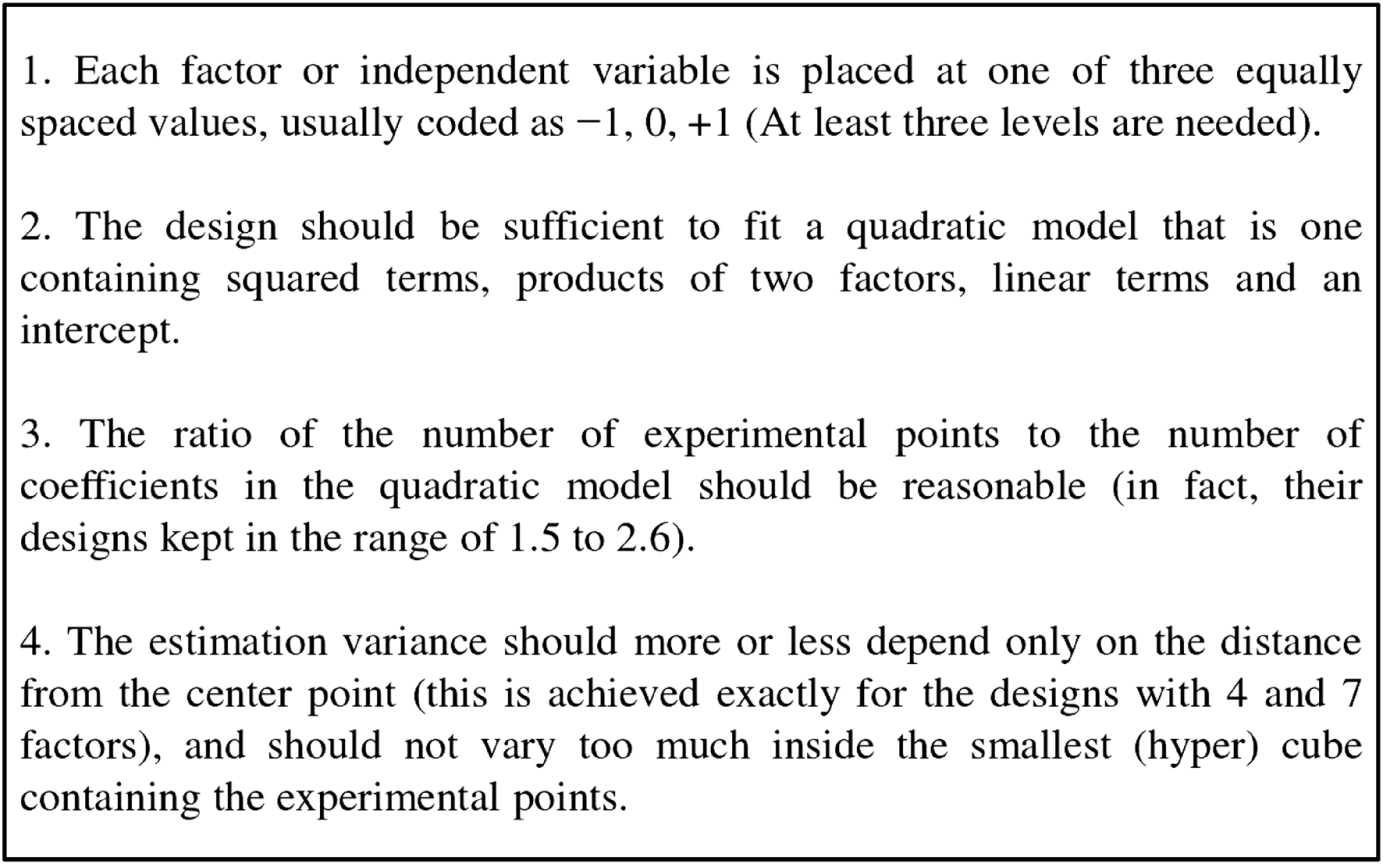
Properties provided for the response surface method (RSM) by George E. P. Box and Donald Behnken.

The response surface method involves constructing experimental models using statistical and mathematical techniques. Box-Behnken designs are employed to develop quadratic models for independent parameters and investigate their effects using fewer runs than traditional factorial techniques. George E. P. developed this method. https://en.wikipedia.org/wiki/George_E._P._BoxIn 1960, Box and Behnken introduced a design with the characteristics depicted in Fig. 2^22^.

The independent and response variables are identified based on the input and output variables. Based on results from previous studies^14,15^ a mathematical model can be created through experimental designs employing factorial design. Box-Behnken design method avoids having all factors at their extremes concurrently, determining coefficients of quadratic polynomial equations via linear regression.

Variance analysis uses interactions between process variables and responses to graphically examine the obtained data. The quality of the adjusted polynomial model is shown by the R^2^ designation coefficients and its statistical significance is represented by the F-test in the same program. Model confidence can beassessed by probability values (P-value) with "95%" confidence limits. 3D plots and their response contours for the rate of FT reaction based on the effects of three variables (partial pressure of carbon monoxide,temperature, and partial pressure of H_2_) are drawn at three levels. The experimental design based on Box–Behnken design is carried out which resulted to 15 experiments to express the rate of FT reaction. The maximum and minimum ranges of the three operation process factors were: the reaction temperature (X_1_) (463.15–503.15 K), carbon monoxide partial pressure (X_2_) (in the 0.9–2.8 bar), and H_2_ partial pressure (X_3_) (0.9–2.8 bar).All these parameters and their properties are tabulated in Table 2. The experiments and responses for kinetic modeling of Co-Mn/SiO_2_ catalyst are summarized in Table 3.

**Table 2:** Designed variables and their properties used in experimental design.

Design Variables (factors)	Coded variables	Actual variables	Actual values of coded levels		
Temperature (K)	X_1_	*T*	463.15	483.15	503.15
Partial pressure of CO (bar)	X_2_	P_CO_	0.9	1.85	2.8
Partial pressure of H_2_ (bar)	X_3_	$P_{{{\text{H}}_{{2}} }}$	0.9	1.85	2.8

**Table 3:** Experimental data for CO consumption over the Co-Mn/SiO_2_ catalyst regard to BBD experimental design method.

No	Factors (input variables)			X_CO_ (%)	Response × 10^3^ (mol g^−1^ min^−1^)
	*T* (K)	*P*_CO_ (bar)	$P_{{{\text{H}}_{{2}} }}$(bar)		
1	463.15	0.9	1.85	0.9	12.632 ± 0.532
2	503.15	0.9	1.85	1.42	18.329 ± 0.981
3	463.15	2.8	1.85	0.73	31.879 ± 1.144
4	503.15	2.8	1.85	1.22	48.922 ± 1.361
5	463.15	1.85	0.9	0.77	22.106 ± 0.875
6	503.15	1.85	0.9	1.13	30.137 ± 1.012
7	463.15	1.85	2.8	0.88	25.319 ± 0.683
8	503.15	1.85	2.8	1.29	34.397 ± 1.125
9	483.15	0.9	0.9	0.89	11.973 ± 0.217
10	483.15	2.8	0.9	0.95	39.943 ± 0.759
11	483.15	0.9	2.8	1.12	15.129 ± 0.067
12	483.15	2.8	2.8	1.09	45.987 ± 1.429
13	483.15	1.85	1.85	1.15	31.675 ± 1.091
14	483.15	1.85	1.85	1.09	30.126 ± 1.481
15	483.15	1.85	1.85	1.06	29.316 ± 0.908

The response surface model with interactions between parameters for the FT reaction rate can be obtained using Eq. (3).

3 $${\hat{\text{y}}} = {\text{b}}_{{0}} + \sum\limits_{i = 1}^{k} {b_{i} } X_{i} + \sum\limits_{i = 1}^{k} {b_{ii} X_{i}^{2} } + \sum\limits_{i < j}^{k} {b_{ij} } X_{i} X_{j}$$

where ŷ is the predicted response, X_i_ is the coded variables, and b_ij_, b_ii_, b_i_, b_0_ are the regression coefficients of the Box–Behnken design method.

## Results and discussion

### Characterization

The BET technique was used to examine the specific surface area, porosity, and the influence of SiO_2_ support on the variations of these properties for the precursor, calcined, and spent nanocatalyst samples. The results are shown in Table 4. As results can be seen that the precursor of catalyst have the highest surface area compared to other. The porosity of precursor varies, indicating that calcination and reaction stages impact sample porosity. The presence of crystallization water and adsorbed physical water in the precursor sample accounts for this. During calcination, the catalyst in the sample released its physical and crystallization water, destroying cavities and resulting in a smaller surface area compared to the precursor.

**Table 4:** BET measurements for nanocatalyst used in FTS reaction.

Catalyst	In case of:	SSA^a^ (m^2^/g)	PV^b^ (cm^3^/g)	MD^c^ (Å)
Co-Mn	Precursor	11.32	1.231 × 10^–1^	16.89
	Before reaction	74.28	2.116 × 10^–1^	13.34
	After reaction	58.88	1.071 × 10^–2^	23.13
Co-Mn/SiO_2_	Precursor	167.282	4.831 × 10^–2^	22.93
	Before reaction	158.2271	3.126 × 10^–2^	23.28
	After reaction	149.341	2.915 × 10^–2^	22.89

^a^Specific surface area.

^b^Pore volume.

^c^Mean diameter.

**Figure 3:**
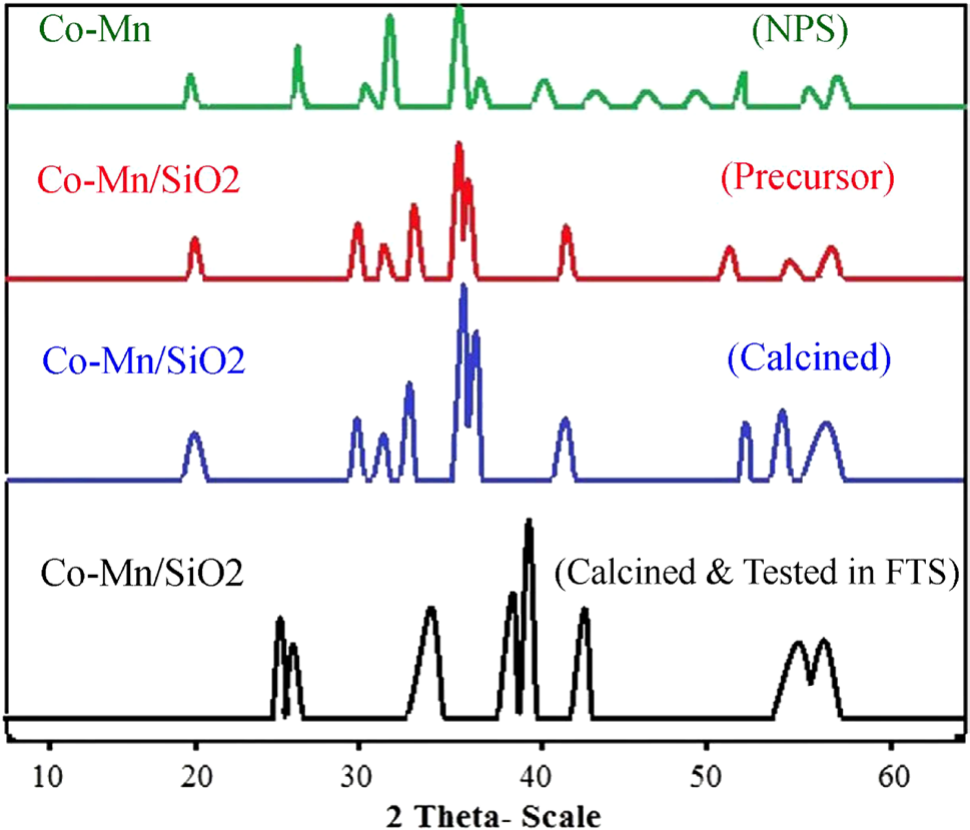
X-Ray analysis for Co-Mn/SiO_2_ nanocatalyst at different states.

The characteristics of the Co-Mn/SiO_2_ catalyst phases in the precursor, before and after the test (H_2_/CO = 2) sampels are depicted in Fig. 3. According to the XRD results, the NPS sample contains the CoMn_2_O_4_ phase, which matches with XRD standard JCPDS data (No.018-0408). The precursor of this catalyst resembles the NPS sample pattern. Incorporation of NPS on SiO_2_ in precursor sample leads to appears of additional peaks at 29°, 33° and 35° indicative Si(OH)_4_. Before the test sample contains different oxide phases including (21°, 31°, 37°, 39°, 44°, 55°, 59°) for Co_3_O_4_ (cubic), (30°, 37°, 43°, 57°, 63°) for MnO_2_ (cubic), CoMn_2_O_4_ (rhombohedral) and (22°, 38.5°, 45°, 59°, 65°) for Co_2_SiO_4_ (cubic). Performing the FT reaction on the surface of this catalyst leads to change and produces different phases such as CoO (cubic) at (30°, 33°, 63°), C (hexagonal) at (10°, 30°, 43°), Co (cubic) at (30°, 43°, 57°) and MnO (cubic) at (30°, 37°, 43°, 57°, 63°). The XRD analysis reveals that the precursor undergoes calcination, resulting in the formation of both oxide and alloy (metallic) phases. After the FT reaction, metal oxides with higher oxidation degrees have been reduced to form lower oxidation degree metal phases.

**Figure 4:**
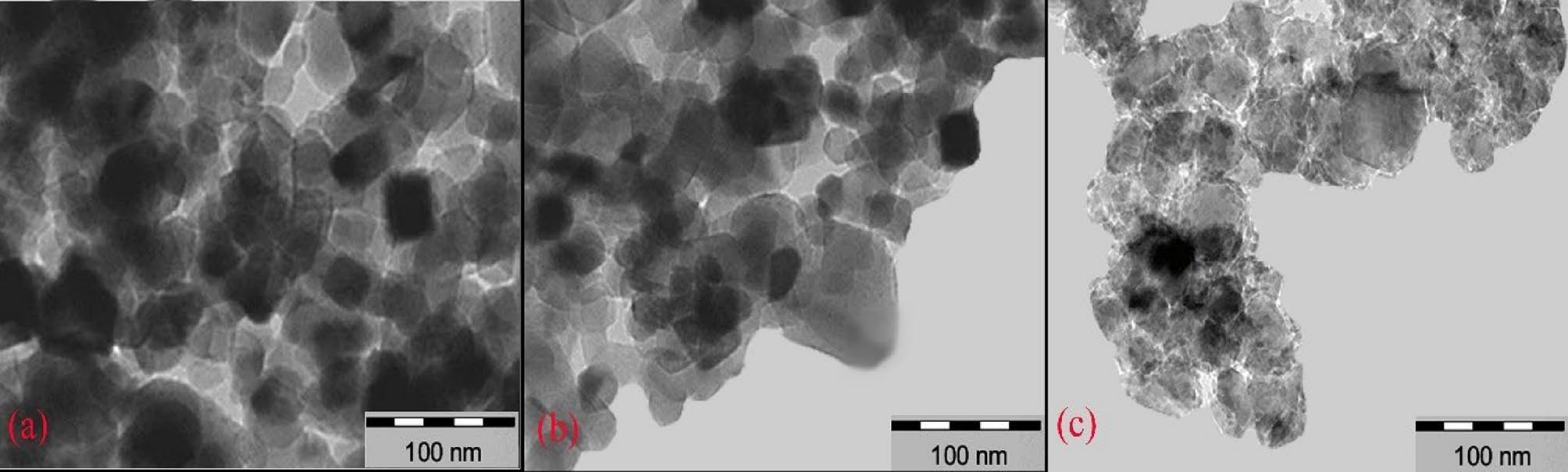
TEM images for Co-Mn/SiO_2_ nanocatalyst in the case of precursor (**a**), calcined (before) (**b**) and after test in FTS (**c**).

The structural changes in the precursor, as determined by XRD analysis, occurred during calcination and continued after the catalytic test. The XRD technique might not accurately measure fine details. Therefore, TEM study was conducted on the precursor and the catalyst before and after the FTS test (at a H_2_/CO ratio of 2), as shown in Fig. 4. Through TEM observations, the precursor and calcined catalyst morphologies have been revealed to differ. Figure 4a illustrates the presence of nonuniform particle agglomerations with reduced density in the taken images. calcinations (Fig. 4b) result in the formation of uniform particles, evident in the morphological change and confirmed by the XRD findings of cobalt and manganese oxide phases in the calcined catalyst sample. The samples size distribution measured to 12 nm. In Fig. 4c, adhesion and aggregation of particles were observed post FTS reaction. The particle size growth in this sample might be justified by cooking occurred during FTS.

### RSM modeling

Using RSM with Design Expert software (version 7, Stat-Ease Inc., USA), multiple regression analysis and optimization processes were carried out, leading to significant analysis of variance (ANOVA) results at *P* < 0.05. The significance of the quadratic models was assessed using analysis of variance. Fischer–Tropsch reaction rate's statistical significance is determined by F values in second-order regression models, which denote the dispersion size of the data from their mean value. The significance of the quadratic model for the FT reaction rate was confirmed with a confidence level of *P* = 0.0002 and F = 56.39 through the analysis of variance of its regression parameters..

The lack of fit parameter was absent here (because 0.2385 is greater than "0.05"), indicating that the quadratic model is valid. In addition, the correlation factor R^2^ = 0.9902 was obtained for the FT reaction, which represented a good match between the calculated and observed results in the experimental range. The final model, which is given in the form of coded parameters and real factors with a quadratic empirical equation can be presented as follows:

4 $$\begin{aligned} R = & - 0.76239 + 0.0031854T - 0.054614P_{{{\text{CO}}}} - 0.0016895P_{{{\text{H}}_{{2}} }} + 0.0001487TP_{{{\text{CO}}}} + 0.00001373TP_{{{\text{H}}_{{2}} }} \\ & \quad + 0.0008P_{{{\text{CO}}}} P_{{{\text{H}}_{{2}} }} - 0.0000033T^{2} - 0.00119865P_{{{\text{CO}}}}^{2} - 0.0011439P_{{{\text{H}}_{{2}} }}^{{2}} \\ \end{aligned}$$

5 $$\begin{aligned} R = & + 30.37 + 4.98X_{1} + 13.58X_{2} + 2.08X_{3} + 2.84X_{1} X_{2} + 0.26X_{1} X_{3} + 0.722X_{2} X_{3} \\ & \quad - 1.35X_{1}^{2} - 1.08X_{2}^{2} - 1.03X_{3}^{2} \\ \end{aligned}$$

**Figure 5:**
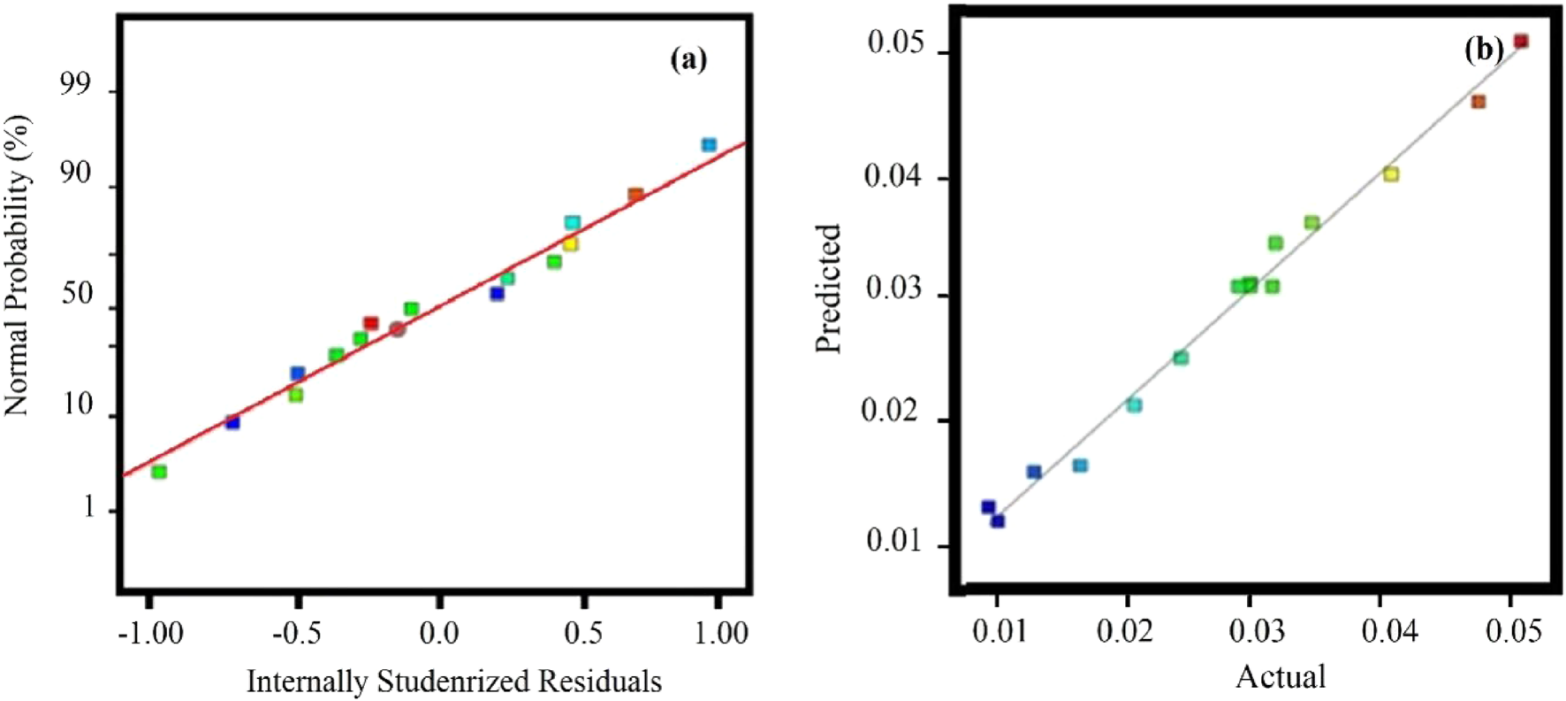
Normal distribution of error (**a**) and compression between experimental and calculated data (**b**) for FT reaction rate.

Figure 5a shows the normal probability of the residuals to check the standard deviation between the actual and predicted response values of a normal distribution. The outcomes presented in Fig. 5a show the general effect of normal distribution of basic errors. Figure 5b presents the actual values based on the predicted values, which shows the data of the actual responses against the predicted responses. The predicted response values slightly deviate from the experimental data.

Comparing the data resulted from different models ("linear", "two-factorial", "quadratic and cubic") and analysis of variance analysis shows that the Fischer–Tropsch reaction rate could best explained by the quadratic polynomial model. Using significance levels of 10, 5 and 1% a model is considered significant if the *P* values (significant probability value) be less than 0.1, 0.05 and 0.001, respectively. From the *P* values offeredin Table 5, it can be concluded that the rate of carbon monoxide consumption in FT reaction is adapted to the linear distribution and vector product distribution of the model, while it was not adapted to the quadratic distribution of the model. It can be seen that there are deviations in applying linear regression and this model has carefully studied the tested range. The most important linear factors such as temperature and carbon monoxide partial pressure are obtained with P values of 0.0006 and 0.0001. The H_2_ partial pressure factor has a *P* value of 0.0249 and is consistent at 5%. On the other hand, the first element of the vector product (T × P_CO_) in 5% corresponds to the value of *P* equal to 0.0285.

**Table 5:** The results of multiple regressions and the significance for RSM model terms.

		Factor	Standard error	*t*-value #	*P*-value	Contributions (%)
Main effects	Linear	$T$	0.66	15.09	0.0006***	11.28
	Linear	$P_{{{\text{CO}}}}$	0.66	41.16	0.0001***	83.92
	Linear	$P_{{{\text{H}}_{{2}} }}$	0.66	6.31	0.0249**	1.97
Interaction	Cross product	$T \times P_{{{\text{CO}}}}$	0.93	6.1	0.0285**	1.83
		$T \times P_{{{\text{H}}_{{2}} }}$	0.93	0.56	0.7896	0.015
		$P_{{{\text{CO}}}} \times P_{{{\text{H}}_{{2}} }}$	0.93	1.55	0.4727	0.12
	Pure quadratic	$T^{2}$	0.97	-2.78	0.222	0.38
		$P_{{{\text{CO}}}}^{{2}}$	0.97	-2.23	0.3147	0.24
		$P_{{{\text{H}}_{{2}} }}^{{2}}$	0.97	-2.12	0.3351	0.22

^#^*t*-value = twice coefficient estimated /standard error.

*significant at 10% (p-value),**Significant at 5% (*P*-value),***significant at 1% (*P*-value).

**Figure 6:**
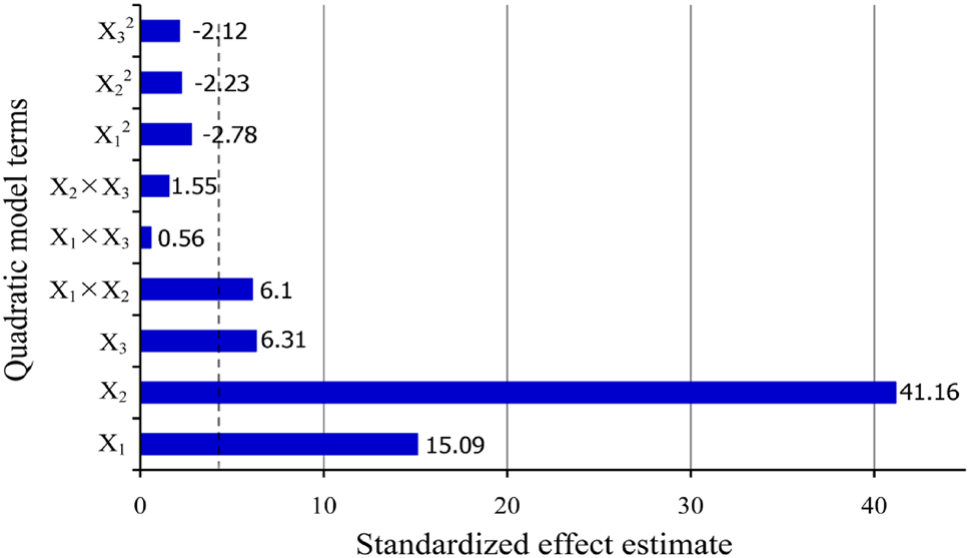
Pareto chart for terms effect in RSM model.

In Fig. 6, Pareto charts illustrate the impact of independent variables and their combined effects. The bar lengths in these graphs indicate the impact of the variables. As can be seen in Fig. 6, the partial pressures of CO (X_2_) has the greatest influence on the FT reaction rate, followed by the temperature (X_1_), the partial pressures of H_2_ (X_3_), and X_1_ÍX_2_ interaction, whereas the X_1_^2^, X_2_^2^, X_3_^2^, X_1_ÍX_3_ interaction, and X_2_ÍX_3_ interaction had no effect. In Pareto charts, negative signs indicate that an increase in the corresponding term results in a decrease in the FT reaction rate due to an antagonistic effect, as observed for X_1_^2^, X_2_^2^, and X_3_^2^. An increase in temperature (X_1_), CO partial pressures (X_2_), and H_2_ partial pressures (X_3_) positively influences the reaction rate.

The percentage contributions of each independent variable in the FT reaction rate are shown in Table 5, calculated using the ANOVA results and the sum of the squares Eq. ^23^:

6 $${\text{Contribution (\% ) }} = \frac{SS}{{\sum {SS} }} \times 100$$

As shown in Table 5, the partial pressures of CO (X_2_) showed the highest effect on FT reaction rate, as its contribution was 83.92%, followed by the temperature (X_1_) at 11.28%, the partial pressures of H_2_ (X_3_) at 1.97%, and X_1_ÍX_2_ interaction at 1.83%, while the other terms exhibited the lowest effect. These results confirm the findings reported in the Pareto charts.

Syngas pressure is one of the important screening parameters in evaluation of catalysts for FT synthesis. Also, changing the overall pressure is used to direct the FT reaction towards the desired products. The results of the FT catalysts at atmospheric pressure are completely different with its efficiencies in high pressure. These differences can be attributed to different concentrations of reagents in liquid and gas phases, catalyst regeneration and its deactivation. In commercial processes, the FT reaction is usually carried out at high pressure. An increase in overall pressure can result in the concentration of hydrocarbons that are normally in a gaseous phase at atmospheric pressure. Higher pressures lead to more carbon monoxide conversion, which may cause the catalyst cavities to become saturated with liquid products. A different composition of the liquid in the catalyst cavities at high pressures of syngas can affect the rate of carbon monoxide and hydrocarbon concentrations. Temperature is a fundamental process variable that has an important effect on the overall yield of the FT reaction and commonly is used to control the distribution of products in a reaction, where one product may predominate at a lower temperature and another at a higher temperature. The reaction temperature also has a great effect on the carbon monoxide conversion rate and the efficiency of the process. All reactions occurring in the FT process are highly exothermic, so controlling of the temperature is essential to ensure that the reaction proceeds to the production of the desired products. Therefore, evaluation the effects of theses parameters and their interactions on the process are essential and can be well presented using 3D- graphs or counters in RSM modeling.

**Figure 7:**
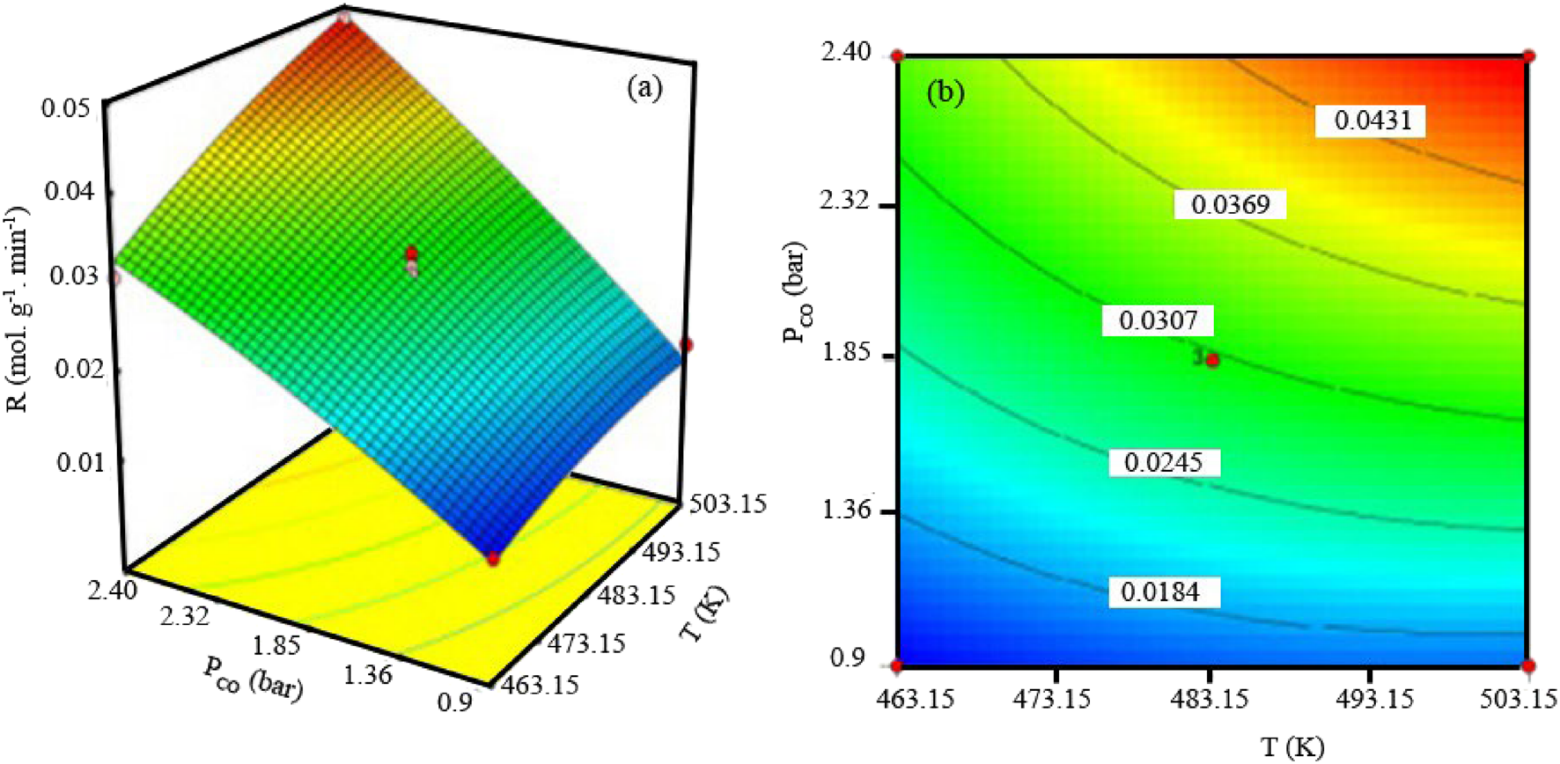
3D graph (**a**) and counter (**b**) for interactions between partial pressure of CO and temperature of reaction.

**Figure 8:**
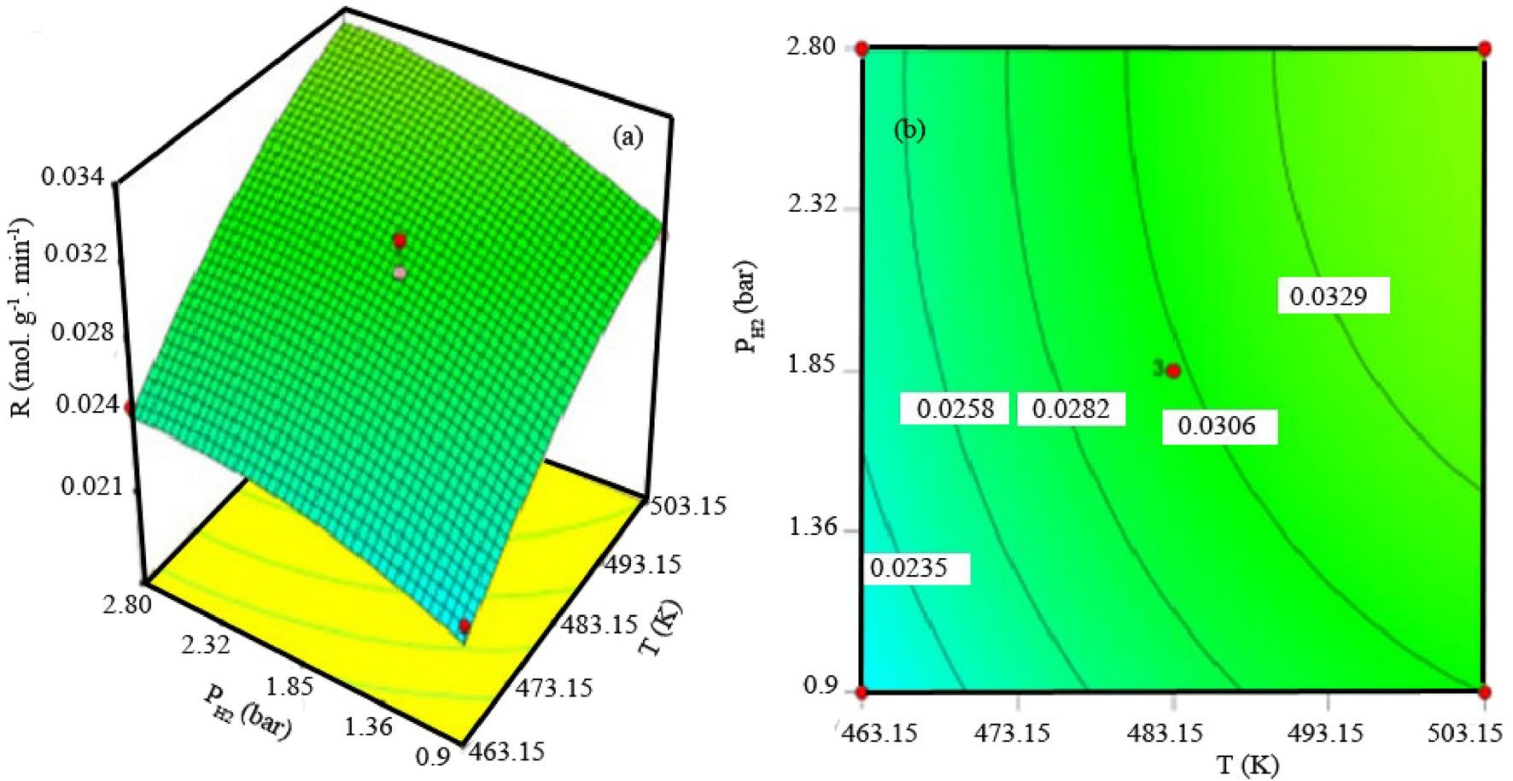
3D graph (**a**) and counter (**b**) for interactions between partial pressure of H_2_ and temperature of reaction.

In Fig. 7, the effects of carbon monoxide partial pressure and temperature on the surface response (reaction rate) were plotted, when H_2_ partial pressure considered constant at 1.85 bar. The existence of interactions between variables of carbon monoxide partial pressure and temperature are evident from both the 3D surface response and the linear contour plot. Increasing the partial pressure of carbon monoxide leads to an increase in the reaction rate, especially at high temperatures. For instance, raising the partial pressure of carbon monoxide at a temperature of 503 K causes the reaction rate to rise from 18.4 × 10^–3^ (mol/g min) to almost 43.15 × 10^–3^ (mol. g min). When the partial pressure of carbon monoxide is raised for lower temperatures, such as 463.15 K, the rate of carbon monoxide consumption only goes up from "18.4 × 10^–3^" to "30.77 × 10^–3^" (mol/g min). Additionally, as the carbon monoxide pressure climbed from 0.9 bar to 2.8 bar, the impact of temperature on the pace of the FT reaction grew. The reaction rate for a constant partial pressure of carbon monoxide at 1.85 bar is shown in Fig. 8 as a function of temperature and partial pressure of H_2_. The rate of carbon monoxide conversion rises when the temperature is raised from 463.15 to 503.15, but only slightly when the partial pressure of hydrogen is raised.

According to the Arrhenius equation, temperature influences the reaction rate constant to a different extent, based on the magnitude of the activation energy. For example, the preference for secondary reactions of the primary FTS products changes with increasing temperature. In this regard, the above approach can change the product selectivity result^24^. Akbarzadeh^25^ depicted that increasing the temperature (from 200 to 280 °C) of the FT reaction, the percentage of CO conversion increases (from 59.5 to 88.2%). The movement of hydrogen on the surface of the catalyst increases as the FTS temperature increases. Therefore, this approach results in higher CO conversion. Due to the increase in temperature and pressure, the increase in the reaction rate is mentioned in the FTS report (in literature^16,21^). Jamala^26^ investigated the increase in CO conversion observed in his study when the operating pressure was increased from 1 to 10 bar to an increase in the pressure of the reactants (H_2_ and CO) in the reactor.

Yang et al.^27^ showed that pressure has a profound effect on how product selectivity changes with CO conversion. They investigated the effects of increasing manganese on the selectivity and activity of 12% Co/SiO_2_ catalyst for FTS using a fixed-bed reactor. Dinseet al.^24^ showed that at 1 bar, the selectivity for all products was unaffected by CO conversion. However, the olefin to paraffin (O/P) ratio of the C_2_-C_4_ fraction decreased with increasing CO conversion due to increased hydrogenation of the olefins with increasing space time. At 10.1 bar, C_2_-C_4_ olefins are included in the growing hydrocarbon chain. This leads to a decrease in C_2_-C_4_ selectivity and an increase in C_5_^+^ selectivity with increasing CO conversion.

**Figure 9:**
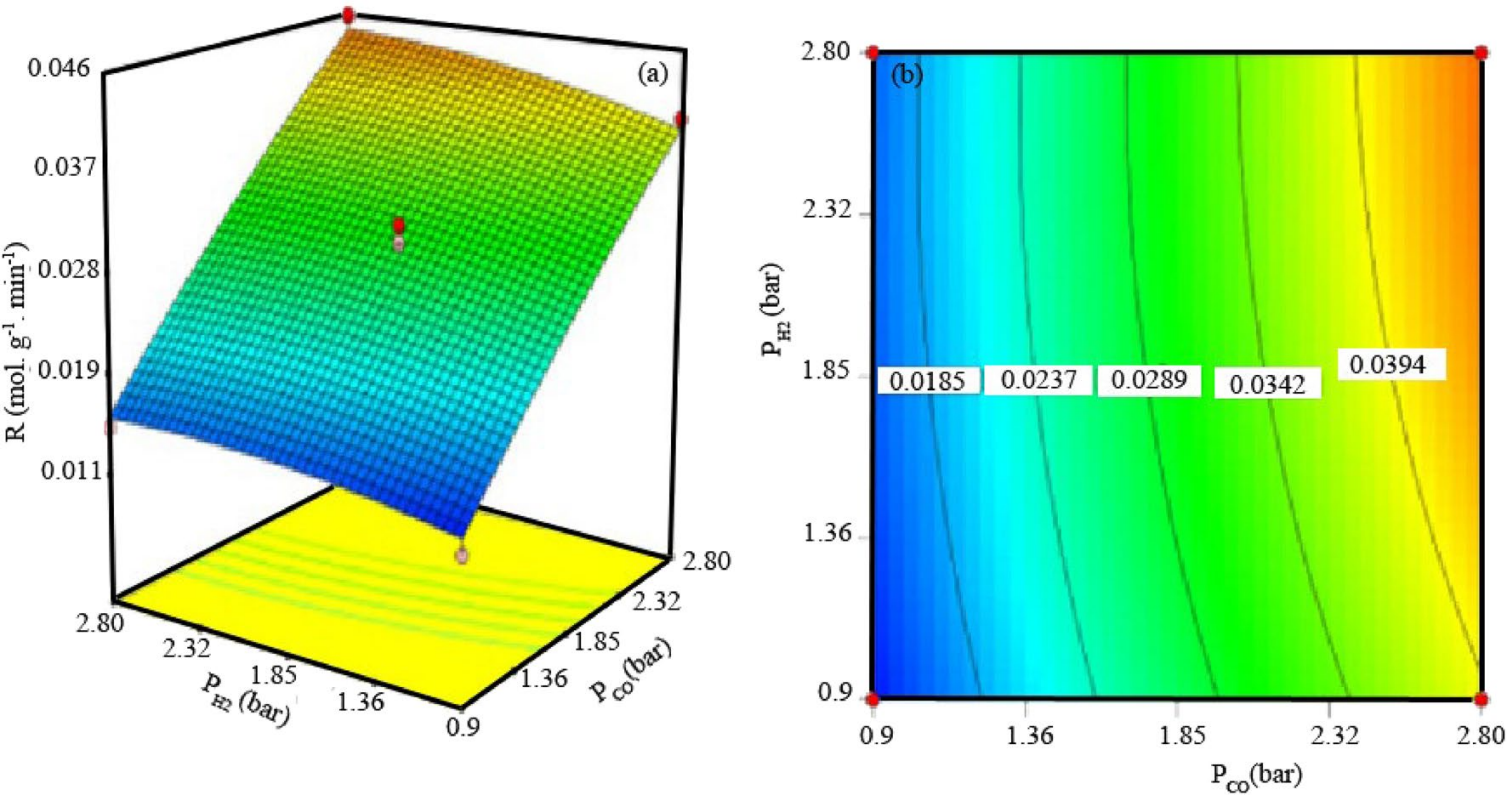
3D graph (**a**) and counter (**b**) for interactions between partial pressures of CO and H2.

In Fig. 9, the surface response and the contour plots show the negligible interactive effects of the partial pressure of hydrogen and carbon monoxide on the reaction rate. Abbaslu et al.^28^presented that with increasing pressure, the supercritical phase displays a liquid-like density that increases the extraction from the catalyst pores. This phenomenon assists to adsorb H_2_ and carbon monoxide on the active sites by increasing the carbon monoxide conversion rate. It has also been stated that the speed of various FT reactions that take place on the catalyst surface are directly affected by the reaction temperature. Like the results of our study, it has been stated that the increase in carbon monoxide conversion is due to the increase in the concentration of carbon radicals on the surface with the increase in pressure, and the probability of collision between the catalysts and the reactants is higher, and therefore the reaction rate increases.

**Figure 10:**
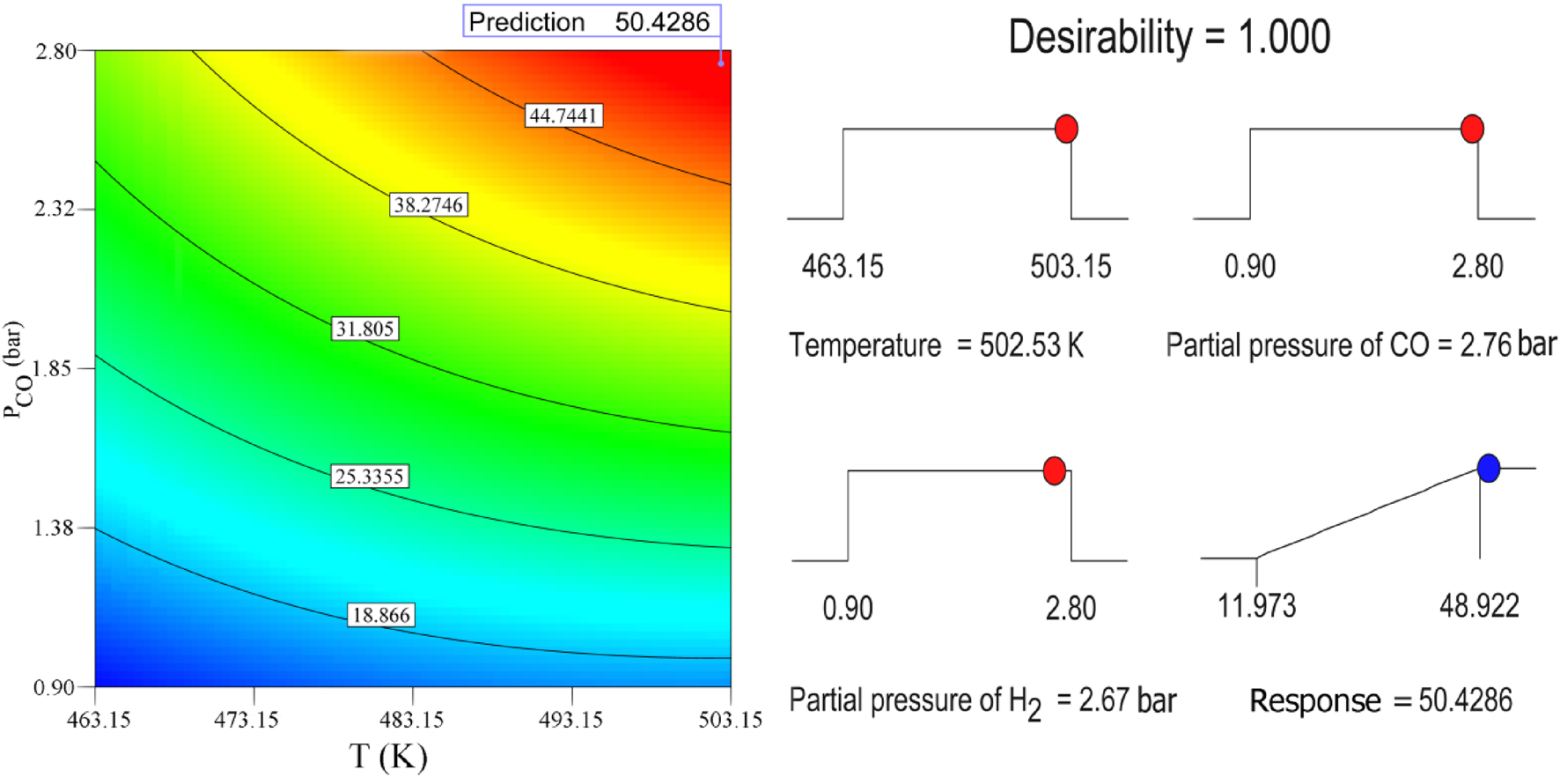
Desirability ramp andContour plot showing optimal condition in terms of response × 10^3^(mol  g^−1^  min^−1^).

In order to choose the optimal conditions to achieve the highest of Fischer–Tropsch reaction rate, temperature range (463.15–503.15 K) and partial pressures of CO & H_2_ (0.9–2.8 bar) is considered. Desirability ramp and two-dimensional contour graph in Fig. 10 shows the rate of CO consumption contour and gained optimal level based on temperature (K) and partial pressure of CO (bar) at partial pressure of H_2_ = 2.67 bar. As can be seen, with the desirability close to 1, one of the optimal points with the highest reaction rate (R = 50.429 × 10^–3^ mol g^−1^ min^−1^) can be determined at temperature of 502.53K and CO partial pressure of 2.76 bar. Under the optimum conditions, the rate of CO conversion reaches to 51.242 × 10^–3^ mol g^−1^ min^−1^ where corresponds to the result obtained by RSM (Error = 1.61%).

### LHHW modeling

To analyze the experimental data and determine the best model of carbon monoxide consumption rate in FT process over the Co-Mn/SiO_2_ nano catalyst, the equations presented in Table 6 were fitted to the data using non-linear regression analysis.

**Table 6:** Kinetic equation for Fischer –Tropsch synthesis reaction rate according to LHHW.

Model	Kinetic equation	Site balance
C-I1	$k\,P_{{{\text{CO}}}} /(1 + a\,P_{{{\text{CO}}}} + b\,P_{{{\text{H}}_{{2}} }}^{{{0}{\text{.5}}}} )$	s + COs + Hs
C-I2	$k\,P_{{{\text{H}}_{{2}} }} /(1 + a\,P_{{{\text{CO}}}} + b\,P_{{{\text{H}}_{{2}} }}^{{{0}{\text{.5}}}} )^{2}$	s + COs + Hs
C-I3	$k\,P_{{{\text{CO}}}} P_{{{\text{H}}_{{2}} }}^{{{0}{\text{.5}}}} /(1 + a\,P_{{{\text{CO}}}} + b\,P_{{{\text{H}}_{{2}} }}^{{{0}{\text{.5}}}} )^{2}$	s + COs + Hs
C-I3′	$k\,P_{{{\text{CO}}}} P_{{{\text{H}}_{{2}} }}^{{{0}{\text{.5}}}} /(1 + b\,P_{{{\text{H}}_{{2}} }}^{{{0}{\text{.5}}}} )^{2}$	s + Hs
C-I4	$k\,P_{{{\text{CO}}}} P_{{{\text{H}}_{{2}} }} /(1 + a\,P_{{{\text{CO}}}} + b\,P_{{{\text{H}}_{{2}} }}^{{{0}{\text{.5}}}} )^{2}$	s + COs + Hs
C-I4′	$k\,P_{{{\text{CO}}}} P_{{{\text{H}}_{{2}} }} /(1 + a\,P_{{{\text{CO}}}} P_{{{\text{H}}_{{2}} }}^{{{0}{\text{.5}}}} )^{2}$	s + HOCs
C-I5	$k\,P_{{{\text{CO}}}} P_{{{\text{H}}_{{2}} }} /(1 + a\,P_{{{\text{CO}}}} )^{2}$	s + COs
C-II1	$k\,P_{{{\text{CO}}}} /(1 + a\,P_{{{\text{CO}}}} + b\,P_{{{\text{H}}_{{2}} }} )$	s + COs + H_2_s
C-II2	$k\,P_{{{\text{H}}_{{2}} }} /(1 + a\,P_{{{\text{CO}}}} + b\,P_{{{\text{H}}_{{2}} }} )$	s + COs + H_2_s
C-II3	$k\,P_{{{\text{CO}}}} P_{{{\text{H}}_{{2}} }} /(1 + a\,P_{{{\text{CO}}}} + b\,P_{{{\text{H}}_{{2}} }} )^{2}$	s + COs + H_2_s
C-II4	$k\,P_{{{\text{CO}}}} P_{{{\text{H}}_{{2}} }}^{{2}} /(1 + a\,P_{{{\text{CO}}}} P_{{{\text{H}}_{{2}} }} )^{2}$	s + CH_2_Os
C-III1	$k\,P_{{{\text{CO}}}} /(1 + a\,P_{{{\text{CO}}}} )$	s + Cos
C-III2	$k\,P_{{{\text{CO}}}} P_{{{\text{H}}_{{2}} }} /(1 + a\,P_{{{\text{CO}}}} )$	s + Cos
C-III3	$k\,P_{{{\text{CO}}}} P_{{{\text{H}}_{{2}} }}^{{2}} /(1 + a\,P_{{{\text{CO}}}} )$	s + Cos
C-III3′	$k\,P_{{{\text{CO}}}} P_{{{\text{H}}_{{2}} }}^{{2}} /(1 + a\,P_{{{\text{CO}}}} P_{{{\text{H}}_{{2}} }} )$	s + CH_2_Os
C-IV2	$k\,P_{{{\text{CO}}}} P_{{{\text{H}}_{{2}} }}^{{{0}{\text{.5}}}} /(1 + a\,P_{{{\text{H}}_{{2}} }}^{{{0}{\text{.5}}}} )$	s + Hs
C-IV3	$k\,P_{{{\text{CO}}}} P_{{{\text{H}}_{{2}} }} /(1 + a\,P_{{{\text{H}}_{{2}} }}^{{{0}{\text{.5}}}} + bP_{{{\text{CO}}}} P_{{{\text{H}}_{{2}} }}^{{{0}{\text{.5}}}} )^{2}$	s + HOCs + Hs
C-V1	$k\,P_{{{\text{H}}_{{2}} }} /(1 + a\,P_{{{\text{H}}_{{2}} }}^{{{\text{o}}{.5}}} )^{2}$	s + Hs
C-V2	$k\,P_{{{\text{CO}}}} P_{{{\text{H}}_{{2}} }} /(1 + a\,P_{{{\text{H}}_{{2}} }}^{{{0}{\text{.5}}}} )^{2}$	s + Hs
C-V3	$k\,P_{{{\text{CO}}}} P_{{{\text{H}}_{{2}} }}^{{2}} /(1 + a\,P_{{{\text{H}}_{{2}} }}^{{{0}{\text{.5}}}} + bP_{{{\text{CO}}}} P_{{{\text{H}}_{{2}} }}^{{{0}{\text{.5}}}} )^{3}$	s + CH_2_Os + Hs
C-VI1	$k\,P_{{{\text{H}}_{{2}} }} /(1 + a\,P_{{{\text{H}}_{{2}} }} )$	s + H_2_s
C-VI2	$k\,P_{{{\text{CO}}}} P_{{{\text{H}}_{{2}} }} /(1 + a\,P_{{{\text{H}}_{{2}} }} )$	s + H_2_s
C-VII1	$k\,P_{{{\text{CO}}}} /(1 + a\,P_{{{\text{CO}}}}^{{{0}{\text{.5}}}} + b\,P_{{{\text{H}}_{{2}} }}^{{{0}{\text{.5}}}} )^{2}$	s + Cs + Os + Hs
C-VII2	$k\,P_{{{\text{H}}_{{2}} }} /(1 + a\,P_{{{\text{CO}}}}^{{{0}{\text{.5}}}} )^{2}$	s + Cs + Os
C-VII2′	$k\,P_{{{\text{H}}_{{2}} }} /(1 + a\,P_{{{\text{CO}}}}^{{{0}{\text{.5}}}} + b\,P_{{{\text{H}}_{{2}} }}^{{{0}{\text{.5}}}} )^{2}$	s + Cs + Os + Hs
C-VII3	$k\,P_{{{\text{CO}}}}^{{{0}{\text{.5}}}} P_{{{\text{H}}_{{2}} }}^{{{0}{\text{.5}}}} /(1 + a\,P_{{{\text{CO}}}}^{{{0}{\text{.5}}}} + b\,P_{{{\text{H}}_{{2}} }}^{{{0}{\text{.5}}}} )^{2}$	s + Cs + Os + Hs
C-VIII3	$k\,P_{{{\text{CO}}}}^{{{0}{\text{.5}}}} P_{{{\text{H}}_{{2}} }} /(1 + a\,P_{{{\text{CO}}}}^{{{0}{\text{.5}}}} + b\,P_{{{\text{H}}_{{2}} }} )^{2}$	s + Cs + Os + H_2_s
C-IX1	$k\,P_{{{\text{CO}}}} /(1 + a\,P_{{{\text{CO}}}}^{{{0}{\text{.5}}}} )^{2}$	s + Cs + Os
C-IX2	$k\,P_{{{\text{H}}_{{2}} }} P_{{{\text{CO}}}}^{{{0}{\text{.5}}}} /(1 + a\,P_{{{\text{CO}}}}^{{{0}{\text{.5}}}} )$	s + Cs + Os
C-X3	$k\,P_{{{\text{CO}}}}^{{{0}{\text{.5}}}} P_{{{\text{H}}_{{2}} }} /(1 + a\,P_{{{\text{CO}}}}^{{{0}{\text{.5}}}} + b\,P_{{{\text{H}}_{{2}} }}^{{{0}{\text{.5}}}} )^{3}$	s + Cs + Os + Hs

The unequal treatment of the values of the kinetic parameters through best-it model (C-III3) was better determined using the Levenberg–Marquardt algorithm by a multivariable nonlinear regression method. In this regard, Eq. (7) was substituted for the reaction rate constant (*k*) and Eq. (8) for the adsorption parameter group (*a*). In kinetic models, Arrhenius and Van't Hoff equations were replaced, respectively:

7 $$k = k_{0} \exp \left( {\frac{ - E}{{RT}}} \right)$$

8 $$a = a_{0} \exp \left( {\frac{{\Delta H_{a} }}{RT}} \right)$$

To minimize the sum of the square of residuals corresponding to difference between the experimental data was the objective function and those calculated for the kinetic models. The R^2^ value (reflects the amount of variance) and root mean square deviation (RMSD) have been reported as measure of the goodness of fit:

9 $$\rho = \frac{1}{{N_{{{\text{exp}}}} }}\sum\limits_{i = 1}^{{N_{{{\text{exp}}}} }} {r_{{\text{CO,i}}}^{{{\text{exp}}}} }$$

10 $$R^{2} = 1 - \left( {\frac{{\sum\limits_{i = 1}^{{N_{{{\text{exp}}}} }} {\left( {r_{{\text{CO,i}}}^{{{\text{exp}}}} - r_{{\text{CO,i}}}^{{{\text{cal}}}} } \right)^{{2}} } }}{{\sum\limits_{i = 1}^{{N_{\exp } }} {\left( {r_{{\text{CO,i}}}^{{{\text{exp}}}} - \rho } \right)^{{2}} } }}} \right)^{{2}}$$

and RMSD is described as:

9 $${\text{RMSD}} = \frac{1}{{N_{\exp } }}\left( {\sum\limits_{i = 1}^{{N_{\exp } }} {\left( {r_{{{\text{CO}},i}}^{\exp } - r_{{{\text{CO}},i}}^{{{\text{cal}}}} } \right)^{2} } } \right)^{2}$$

$r_{{\text{CO,i}}}^{{{\text{exp}}}}$ and $r_{{\text{CO,i}}}^{{{\text{cal}}}}$ show the experimental and calculated CO conversion rate obtained from each kinetic model in *i*th data point, and *N*_exp_ explain the number of experimental data points with pure error variance $\rho$. To select the most suitable kinetic expression, different statistical indices can also be used to determine the quality of regression models via the Polymath software 6.0.

The values of kinetic parameter estimation and statistically indicator for each model are presented in Table 7. Based on the statistical criteria and by comparing the values of R^2^ and RMSD it was recognized that C-III3 is the most appropriate model. The obtained values for the constants of the optimal equation are greater than the 95% confidence level. The amount of variance (deviation from standard) of calculation is almost zero for the optimal equation and its correlation coefficient is higher than 96%, which all confirm the appropriateness of the model with the obtained coefficients. The other models were discarded due to the negative coefficients and small value of R^2^ and the large number of 95% confidence of the obtained value for the parameters.

**Table 7:** Kinetic and statistical parameters for each LHHW equation resulted from nonlinear regression fitting.

Model	Kinetic parameter			Statically indicator		
	k (x) (mol g^−1^ min^−1^ bar^x^)	$a$(x) (bar^x^)	b (x) (bar^x^)	R^2^	RMSD	Variance
C-I1	0.000901 (− 1)	− 0.128 (− 1)	− 0.0788 (− 1/2)	0.987	0.00029	1.37E−06
C-I2	0.000673 (− 1)	− 0.118 (− 1)	− 0.709 (− 1/2)	0.978	0.00044	3.14E−06
C-I3	0.000614 (− 3/2)	− 0.00748 (− 1)	− 0.852 (− 1/2)	0.981	0.00040	2.63E−06
C-I3′	0.00064 (− 3/2)	− 0.859 (− 1/2)		0.981	0.00040	2.37E−06
C-I4	0.000935 (− 2)	0.00972 (− 1)	− 0.842 (− 1/2)	0.979	0.00043	2.96E−06
C-I4′	0.00541 (− 2)	− 0.310 (− 3/2)		0.784	0.00139	2.81E−05
C-I5	0.0146 (− 2)	− 0.0367 (− 1)		0.746	0.00151	3.31E−05
C-II1	0.00124 (− 1)	− 0.211 (− 1)	− 0.673 (− 1)	0.99	0.00029	1.42E−06
C-II2	0.00195 (− 1)	− 0.293 (− 1)	− 0.547 (− 1)	0.984	0.00037	2.31E−06
C-II3	0.00216 (− 2)	− 0.0129 (− 1)	− 0.723 (− 1)	0.981	0.00041	2.69E−06
C-II4	0.00168 (− 3)	− 0.542 (− 2)		0.964	0.00056	4.66E−06
C-III1	0.00574 (− 1)	− 0.31 (− 1)		0.519	0.00208	6.28E−05
C-III2	0.0148 (− 2)	− 0.061 (− 1)		0.746	0.00151	3.31E−05
C-III3	0.0224 (− 3)	0.0587(− 1)		0.962	0.000535	4.12E−06
C-III3′	0.0064 (− 3)	− 0.523 (− 1)		0.993	0.00022	7.59E−07
C-IV2	0.00224 (− 3/2)	− 0.918(− 1/2)		0.99	0.00026	1.04E−06
C-IV3	0.06 (− 2)	− 0.897 (− 1/2)	0.247 (− 3/2)	0.99	0.00019	6.27E−07
C-V1	0.00176 (− 1)	− 0.787 (− 1/2)		0.947	0.00064	5.82E−05
C-V2	0.00163 (− 2)	− 0.746 (− 1/2)		0.994	0.00020	5.87E−05
C-V3	0.00346 (− 3)	− 0.436(− 1/2)	− 0.0312 (− 3/2)	0.994	0.00020	6.75E−07
C-VI1	0.0057 (− 1)	− 0.863 (− 1)		0.92	0.00076	8.31E−06
C-VI2	0.0050 (− 2)	− 0.812 (− 1)		0.99	0.00076	7.66E−07
C-VII1	0.00038 (− 1)	− 0.165 (− 1/2)	− 0.682 (− 1/2)	0.996	0.00015	3.94E−07
C-VII2	0.00342 (− 1)	− 0.502 (− 1/2)		0.819	0.00117	1.97E−05
C-VII2′	0.00058 (− 1)	− 0.321 (− 1/2)	− 0.489 (− 1/2)	0.996	0.00017	4.651E−07
C-VII3	0.00048 (− 1)	− 0.244 (− 1/2)	− 0.584 (− 1/2)	0.996	0.00015	4.04E−07
C-VIII3	0.0012 (− 3/2)	− 0.332 (− 1/2)	− 0.400 (− 1)	0.996	0.00015	4.05E−07
C-IX1	0.00264(− 1)	− 0.416 (− 1/2)		0.65	0.00163	3.82E−05
C-IX2	0.007 (− 3/2)	− 0.519 (− 1/2)		0.834	0.00112	1.82E−05
C-X3	0.000435 (− 3/2)	− 0.0853 (− 1/2)	− 0.671 (− 1/2)	0.977	0.000456	3.33E−06

The optimal kinetic model obtained for Co-Mn nanocatalyst supported by silica is based on the reaction of molecular hydrogen with adsorbed carbon monoxide and the formation of methylene and then methyl monomers. The rate-determining step is the reaction of adsorbed methylene with adsorbed hydrogen atom and the most active species on the surface of the catalyst is only carbon monoxide.

Table 8 shows the activation energy and calculated kinetic parameters for the best fitted model (C-III3). With the Arrhenius equation, the activation energy of a reaction is obtained under different conditions. Table 9 summarizes activation energy for the proposed model (C-III3) and literature published values. For the overall FT reaction, the activation energy is mostly between 30 kJ/mol and 110 kJ/mol^6–20,24–28^. In the current study, the activation energy value was estimated to be 61.06 kJ/mol, which is close to activation energy reported previously 62 kJ/mol by Zohdi-Fasaei etal.^17^. The amount of activation energy reported for the formation of hydrocarbons shows that the reports presented in the experiments regarding diffusion interference are not considerable. The high activation energy for hydrocarbon formation suggests that the diffusion interference is not significant in the experiments. As with intraparticle diffusion limitations, the presence of external mass-transfer limitations could be detected via measuring the apparent activation energy. In general, the external mass transfer control regime that can lead to apparent activation energy is only a few kJ/mol^15^.

**Table 8:** Values of kinetic parameters of C-III3 model.

Parameter	Dimension	Estimate
*k*_0_	mol g^−1^ min^−1^ bar^−3^	14.15E+04
*E*	kJ/mol	61.06
$a_{{0}}$	bar^−1^	2.43E+03
$\Delta H_{{\text{a}}}$	kJ/mol	− 26.32

**Table 9:** Comparison of different activation energy from different cobalt based catalyst.

Catalyst	Ea (Kj mol^−1^)	References
This work	61.06	–
La.Fe_0.7_.Co_0.3_	106.25	^10^
Industrial cobalt based	80.26	^11^
Co-Sn/γ-Al_2_O_3_	31.69	^12^
Co-Ni/SiO_2_	98.5	^16^
Co-Mn-Ce/SiO_2_	62.00	^17^
Co-Re/ γ-Al_2_O_3_	92.00	^18^
Ru–Co@C(Z-d)@void@CeO_2_	73–92	^28^

## Conclusion

Co-Mn nanoparticles synthesized through the thermal decomposition of [Co (NH_3_)_4_CO_3_] MnO_4_ complex (incorporated on SiO_2_) and used as catalyst for FT synthesis. The experimental design and the response surface method were employed to create a quadratic model that depicts FT reaction rate in syngas conversion. Based on the results of the experiments, a quadratic polynomial equation was developed to represent the empirical relationship between the response (reaction rate) and the independent variables (temperature and the partial pressures of carbon monoxide and hydrogen). The investigation into how these operational variables affect the FT reaction rate revealed that the rate of carbon monoxide consumption has a significant impact on the reaction conditions. The response surface and contour plot of the anticipated model responses provide confirmation of the influence of the experimental conditions on the rate of carbon monoxide consumption in the FT reaction. The outcomes demonstrated that carbon monoxide's temperature and partial pressure played a significant influence, and that the reaction rate increased with rising pressure and temperature. The response surface model was confirmed by analysis of variance after the analysis of variance revealed R^2^ = 0.9902. Therefore, the model can be used to mechanistically investigate the effect of process variations, syngas composition, temperature and pressure, on the FTS process modeling. To more verification, the reaction rate of FT evaluated using LHHW method and 33 equations tested to fit the experimental data by nonlinear regression method. The rate-determining step involved the reaction of adsorbed methylene and hydrogen atoms, while the most active surface species was carbon monoxide. According to previous research with varied cobalt-based catalysts, our findings using the Co-Mn/SiO_2_ nanocatalyst align, featuring activation energy of 61.06 kJ/mol. This model for FTS utilizing the cobalt-based catalyst effectively predicted the experiment's results, particularly capturing the intricate response of reaction rate towards temperature and pressure. The results clearly demonstrated that the hybrid RSM/LHHW approaches are promising for comprehensive modeling and optimization of a catalytic system in FTS.

## Data Availability

The datasets generated during and/or analysed during the current study are available from the corresponding author on reasonable request.
